# Computational research of mTORC1 inhibitor on cerebral ischemia-reperfusion injury

**DOI:** 10.18632/aging.203371

**Published:** 2021-08-03

**Authors:** Hui Li, Wenzhuo Yang, Zhenhua Wang, Xu Wang, Yulei Hao, Jianxin Xi, Han Lu, Zhishan Du, Jiachun Feng, Bao Zhang, Di Ma

**Affiliations:** 1 Department of Neurology and Neuroscience Center, The First Hospital of Jilin University, Changchun, China; 2 Department of Urology Surgery, Aerospace Center Hospital, Beijing, China; 3 Clinical College, Jilin University, Changchun, China

**Keywords:** cerebral ischemia/reperfusion injury (CIRI), the mammalian target of rapamycin (mTORC1), mTORC1 inhibitors, autophagy

## Abstract

Ischemic stroke contributes to more than 80% of all strokes and has the four characteristics of high prevalence, high disability, high mortality, and high recurrence. Stroke is a preventable and controllable disease. In addition to controlling the primary disease, effective prevention and control measures need to be given to the occurrence and development of stroke. With the development and progress of modern treatment methods for ischemic stroke, the mortality and disability rate have decreased significantly. At present, the main treatment methods for ischemic stroke include thrombolysis, thrombus removal at the ultra-early stage, and treatment of improving collateral circulation in the acute phase. However, the ultra-early and early blood reperfusion involves reperfusion injury, which will cause secondary nerve damage, which is called cerebral ischemia/reperfusion injury (CIRI). Studies have found that autophagy is involved in the entire process of CIRI and can reduce the damage of CIRI. The mammalian target of Rapamycin (mTORC1) is the primary signal pathway regulating autophagy. And the mTORC1 inhibitor, Rapamycin, has been proved to exert neuroprotective effects in the ultra-early and early cerebral ischemia-reperfusion. Therefore, screening and designing mTORC1 inhibitors is very important to control reperfusion injury and reduce neuronal death and apoptosis. In this research, plenty of computer-assisted was applied to virtually screen and select potential mTORC1’s inhibitors. We used Libdock to screen the structure and performed toxicity predictions, ADME (absorption, distribution, metabolism, excretion) to predict small molecules’ pharmacological and toxicological properties. To assess the binding mechanism and affinity between the mTORC1 dimer and the ligand, molecular docking was performed. Then, the pharmacophore of small molecules in the docking conformation with the protein was supplemented by Schrodinger. Additionally, molecular dynamics simulations were conducted to assess if the ligand-receptor complex was stable in a natural environment. Furthermore, an experiment was performed to verify the inhibitory effect of compound 1 and compound 2 on mTOR protein. All in all, the study provides a hand of candidate drugs as well as pharmacological properties, which can play an essential role in mTORC1 inhibitors.

## INTRODUCTION

Ischemic stroke accounts for the highest proportion of all strokes and has the four characteristics of high prevalence, disability, mortality, and recurrence rate. Stroke is a preventable and controllable disease. In addition to controlling the primary disease, effective prevention and control measures need to be given to the occurrence and development of stroke. As modern treatments such as thrombolysis and thrombus removal in the ultra-early stage of ischemic stroke and the treatment of improving collateral circulation in the acute phase have made rapid progress, the mortality and disability rate of stroke have dropped significantly. However, the early and ultra-early blood reperfusion involves reperfusion injury, which will cause secondary nerve damage, which is called cerebral ischemia/reperfusion injury (CIRI). Ischemia-reperfusion injury means the main factor causing damage to the tissue, not the ischemia itself. What damages tissue most is the attack of excessive free radicals on cells after the blood supply is restored. Thus, CIRI is an essential factor that aggravates the pathophysiological process of cerebral ischemia prognosis.

CIRI involves a complex waterfall chemical cascade with multiple levels, multiple processes and multiple targets. And various pathological changes were also involved, such as oxidative stress, hypertension, autophagy, aging death and endoplasmic reticulum stress [1]. The diseased tissue can be divided into the ischemic central area and the penumbra area. The degree of ischemia in the central area is the most serious, and neurons are rapidly necrotic. The surrounding penumbra area is light in ischemia, but the neuronal function is inhibited. It is the main area that we need to save after ischemic stroke. Through timely drug thrombolysis or mechanical thrombectomy within the time window, timely recanalization of cerebral blood flow is the best treatment for ischemic stroke. However, early and ultra-early blood reperfusion will cause CIRI to neurons in the penumbra. Thus, using appropriate methods to control reperfusion injury will reduce neuronal death and apoptosis and effectively improve the functional recovery of patients with cerebral ischemia. Moreover, studies have found that autophagy is involved in the entire process of CIRI [2]. The mammalian target of Rapamycin (mTORC1) is the primary signal pathway regulating autophagy. And the mTORC1 inhibitor, Rapamycin, has been proved to exert neuroprotective effects in the ultra-early and early cerebral ischemia-reperfusion [2]. So, screening and designing mTORC1 inhibitors is very important to control reperfusion injury and reduce neuronal death and apoptosis. In addition, although some existing drugs have been shown to reduce ischemia and hypoxia damage and exert neuroprotective effects in animal models and *in vitro* experiments, they are clinically ineffective against ischemic stroke. So, developing new treatment methods or drugs targeting the autophagy pathway is particularly important for reducing and treating CIRI [3].

Moreover, autophagy is composed of macro-autophagy, micro-autophagy and chaperone-mediated autophagy [4]. Since it is believed that macro-autophagy is the primary means of cytoplasm to lysosome delivery, the term “autophagy” will be used herein to refer to the process of macro-autophagy. The process of autophagy includes signal stimulation, formation of phagocytic vesicles, the fusion of phagocytic vesicles with inclusion bodies/lysosomes, degradation of contents and release of degradation products. In addition, mTORC1 is a crucial protein in the PI3K/AKT/mTORC1 autophagy signaling pathway [5, 6]. And mTORC1 plays an inhibitory role in the formation of phagocytic cysts [7]. In yeast, the formation of phagocytic vesicles requires autophagy-related protein 1(Atg1) and autophagy-related protein 13(Atg13) to form a complex, and the formation of this complex is regulated by the energy-sensitive protein TOR kinase. When the cells are adequately nourished, mTORC1 kinase activates and catalyzes the phosphorylation of Atg13, thereby preventing it from forming a complex with Atg1. Then the formation of phagocytic vesicles [8]. Conversely, when cells are starved or hypoxic, mTORC1 kinase loses activity. Unphosphorylated Atg13 and Atg1 form a complex. The complex then promotes the formation and expansion of phagocytic vesicles. In mammals, Ulk-1 or Ulk-2 replaces Atg1’s function.

Furthermore, as an adaptive cellular response, autophagy is a mechanism to maintain cell homeostasis by removing misfolded proteins and damaged organelles so that cells can avoid apoptosis. When autophagy is not enough to support cell survival, cells will initiate apoptosis, thus ensuring controllable and effective removal of cells without causing local inflammation. However, in the early stage of CIRI, insufficient autophagy leads to excessive cell apoptosis, and local inflammation aggravates nerve damage. Additionally, mTORC1 inhibitors were reported to prevent anti-apoptotic signals, thereby stimulating autophagy and inhibiting apoptosis from exerting neuroprotective effects [9, 10]. What’s more, mTORC1 inhibitors can inhibit microglial activation and reduce the release of neuroinflammatory mediators, which will protect the penumbra after CIRI from secondary damage [11, 12]. Thus, screening and designing mTORC1 inhibitors is quite significant for the treatment of CIRI [13, 14].

In addition, the domain of mTORC1 is composed of HEAT sequence, FRB sequence (rapamycin binding site), kinase domain (K.D.) and FAT-C terminal (FATC) from amino to carboxyl-terminal. Rapamycin can bind to FKBP12 (FK506-binding protein12) and inhibit mTORC1, thereby activating autophagy and immunosuppression. For this reason, Rapamycin was selected as the reference molecule for mTORC1 inhibitors.

Recently, the discovery of natural products has made significant contributions to both molecular biology research and potential drug development. Firstly, virtual screening was conducted through the N.P. (Natural Products database) in the ZINC database to discover new potential mTORC1 inhibitors. Then, the absorption, distribution, metabolism, excretion (ADME) and toxicity of the molecule were analyzed. Through docking, the interaction between potential compounds and mTORC1 was also assessed. Then, the pharmacophore of small molecules in the docking conformation with the protein was supplemented by Schrodinger. Additionally, molecular dynamics simulations were carried out to analyze the stability of binding interactions. Finally, an experiment was performed to verify the inhibitory effect of compound 1 and compound 2 on mTOR protein**.** All in all, this research provides many potential inhibitor drugs and their pharmacological properties, which will significantly promote the development of mTORC1 inhibitor drugs.

## MATERIALS AND METHODS

### Software for docking and ligand database

Discovery Studio is a new molecular modeling environment on a personal computer, professional life science molecular simulation software [15]. According to the structure and biochemical characteristics, Discovery Studio was used to screen, design, and modify potential drugs. With this method, a large number of candidate drugs and lead compounds have been identified and refined. Firstly, we use Libdock, ADME (absorption, distribution, metabolism, excretion) and TOPKAT (Toxicity Prediction by Computer Assisted Technology) modules of DS4.5 (Discovery Studio 4.5 software, Accelrys, Inc.) to accomplish the virtual screening. And then, CDOCKER module was applied for precise docking research. In addition, Schrodinger is a complete software package for drug discovery, including docking modes of receptors and ligands under various conditions, pharmacophore analysis, biomolecular structure simulation, ADME property prediction, etc. So, we chose it to verify the docking results made by DS 4.5. Moreover, Small molecules were downloaded from the ZINC15 database, a free commercially available compound database offered by Irwin and Shoichet Laboratories of the Department of Medicinal Chemistry at the UCSF (University of California, San Francisco, CA, USA) [16].

### Virtual screening based on the structure using libdock

**Figure 1:**
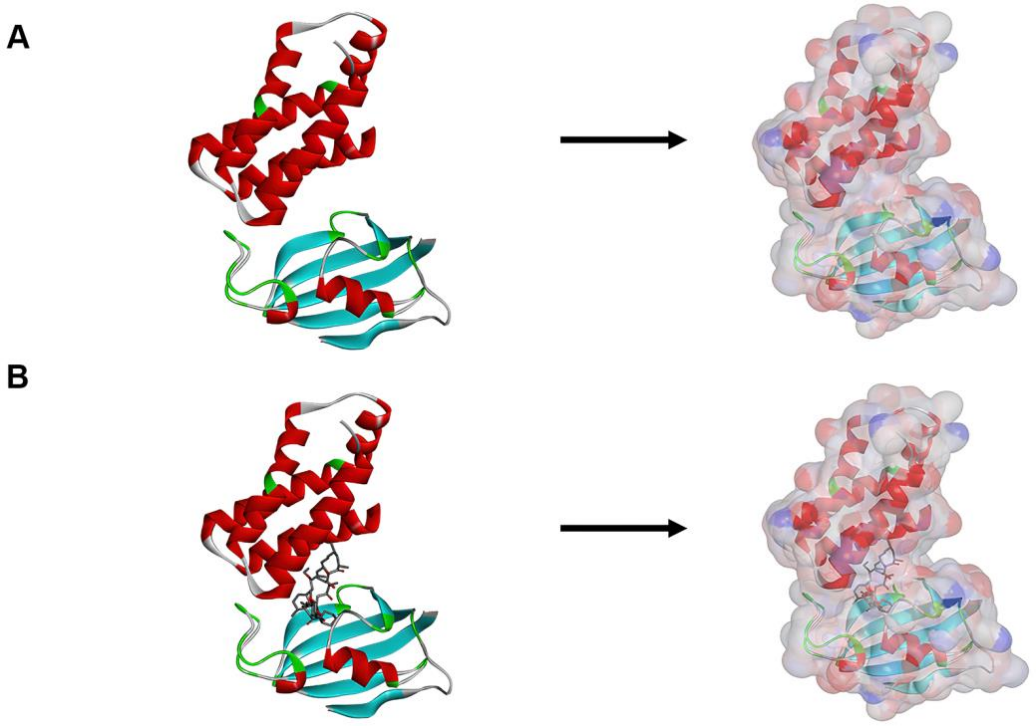
(**A**) The molecular structure of mTORC1. Initial molecular structure was shown, and the surface of the molecule was added. (**B**) The complex structure of mTORC1 with Rapamycin. Initial complex structure was shown, and the surface of the complex. was added. Blue represented positive charge, red represented negative charge.

Firstly, to find new compounds that may restrain mTORC1, we chose the binding pocket of mTOR protein and Rapamycin as the docking site. Furthermore, the diameter of the selected binding sphere was similar to the size of the binding pocket. And we set the 13 Å as the active site diameter according to the PDB site records. The hot spots of the protein were calculated by placing a grid at the binding site and using non-polar and polar probes. The hot spots were then applied to arrange the ligands to interact favorably. The CHARMm force field (Cambridge, MA, USA) and Smart Minimiser algorithm were also carried out to achieve the ligands minimization. Then we ranked all the poses following the scores of ligands after minimization. The 3.22 Å crystal structure of FRB sequence (rapamycin binding site) of mTORC1 in complex with Rapamycin was downloaded from PDB (protein data bank) and then applied to Libdock. Figure 1 displayed the 3D structure of mTORC1’s FRB sequence. A few operations need carrying out when the protein was prepared, including removing crystal water and other heteroatoms, hydrogenation, ionization, protonation and minimization of energy. Additionally, we apply the Smart Minimiser algorithm and CHARMm force field to minimize energy [16].

### ADME (absorption, distribution, metabolism, excretion) and prediction of toxicity

The ADME (Absorption, Distribution, Metabolism, Excretion) of selected molecules [17] were all calculated by DS 4.5. TOPKAT (Toxicity Prediction by Computer Assisted Technology) modules of DS 4.5 also play a vital role in evaluating the toxicity and other properties of all the potential compounds. The analysis of these two modules consists of their aqueous solubility, cytochrome P450 2D6 (CYP2D6) inhibition, plasma protein binding (PPB) level, blood-brain barrier (BBB) penetration, hepatotoxicity, human intestinal absorption, rodent carcinogenicity, AMES mutagenicity, rodent carcinogenicity and developmental toxicity potential [18]. Among them, plasma protein binding rate refers to the ratio of the amount of plasma protein binding to the total blood dose after the drug enters the blood. Generally, protein whose binding rate is high eliminated slowly in the drug body. The effect maintains a long time and stably. On the contrary, the drug with a low binding rate eliminates quickly in the body, and the effect has a massive fluctuation. Additionally, TOPKAT modules quickly and accurately calculate and verifies the toxicity and environmental effects of compounds based on 2D molecular structure. The process uses a series of powerful and cross-validated quantitative structural toxicity relationship (QSTR) models to evaluate different toxicity prediction results. When selecting drug candidates for mTORC1, all pharmacological properties above were considered.

### More precise molecular docking and pharmacological analysis

Based on CHARMm36 force field, CDOCKER module was used for precise docking study between molecules and mTORC1 protein. The receptor remained rigid, while the ligand could be flexible during the docking process. The interaction energy and CHARMm energy (interaction energy plus ligand strain) reflecting ligand binding affinity were also within our calculation for each complicated pose. The crystal structure of mTORC1’s FRB sequence was obtained from the PDB. Considering that the fixed water molecules might affect the formation of receptor-ligand complex, crystal water molecules were generally removed in the rigid and semi-flexible docking process. Then, the water molecules were removed and followed by the addition of hydrogen atoms to the protein. Moreover, the initial compound Rapamycin was firstly extracted from the binding site and then re-docked into the crystal structure of mTORC1 to prove that the combination model was reliable. Then, CHARMm36 force field was applied for both ligands and receptors. The binding site sphere of mTORC1 was defined as the region that came within radius 13 Å from the geometric centroid of the ligand Rapamycin. During the docking process, the residues within the binding site spheres and ligands would interact and combine gradually. After being prepared, structures of identified hits were docked into the binding pocket of mTORC1. Afterward, we performed the CDOCKER process. Each ligand generated ten docking poses, and the best pose was chosen according to the appropriate docking direction and high docking score [19, 20]. Based on CDOCKER interaction energy, the different postures of each test molecule were generated and assessed separately. Additionally, to make the results more credible, carried out by CDOCKER, the procedure was crosschecked again with Schrodinger.

What’s more, the pharmacophore of small molecules in the docking conformation with the protein was performed by Schrodinger. In this procedure, multiple feature pharmacophores are analyzed, such as hydrogen acceptor, hydrogen donor, hydrophobic center and aromatic ring.

### Molecular dynamics simulation

Among the poses predicted by the molecular docking program, the best ligand-mTORC1 complex binding conformation was selected, and then molecular dynamics simulations were performed. The ligand-receptor complex was placed in an orthorhombic box and solvated using an explicit periodic boundary solvated water model. Then sodium chloride was added to the system with the ionic strength of 0.145 to simulate the physiologic environment. Afterward, we subjected the system to the CHARMM force field and relaxed it through energy minimization (500 steps of conjugate gradient and 500 steps of steepest descent). And the final root means square gradient was 0.227. Then, the system’s temperature was slowly driven from an initial temperature of 296 K to the aimed temperature of 302 K within 2 ps. The time of equilibration simulations was 5 ps. Molecular dynamics simulation (production module) lasted for 25 ps with 1 fs time step. We completed the simulations under the normal pressure and the relatively constant temperature of nearly 300 K throughout the procedure. The particle mesh Ewald algorithm was applied for the calculation of long-range electrostatics. And the linear constraint solver algorithm was adapted to identify all bonds involving hydrogen. Taking the initial complex settings as a reference, the structural features, potential energy, and trajectory of RMSD were determined by analyzing DS 4.5’s trajectory module.

### Experiment to verify the inhibitory effect of compound 1 and compound 2 by establishing the enzymatic reaction system and determining mTOR protein activity

### Experimental reagents and supplies

mTOR protein (bought from Wuhan Huamei Biological Company), Atg13 (bought from Shanghai Kemin Biological Technology Co., Ltd.), ZINC000013374324: Aurantiamide Acetate (CAS No.: 56121-42-7; bought from MedChemExpress) and ZINC000012495776: Ltb4 Ethanol Amide (CAS No.: 877459-63-7; bought from Good Laboratory Practice Bioscience). High-performance liquid chromatography (LISPHER100 RP18E 5MYM CART250-4, bought from Supelco).

### Establishment of the enzymatic reaction system and determination of mTOR protein activity

Firstly, we prepared a series of concentration drugs: 10 nmol/L ~0.1mmol/L. Then, different concentrations of drug 1 and drug 2 solutions were added to the environment containing the mTOR protein and its substrate Atg13 protein. Detected by High-performance liquid chromatography (LISPHER100 RP18E 5MYM CART250-4, bought from Supelco), the concentration of substrate under different conditions was determined.

## RESULTS

### Virtual screening of natural products database against inhibitors of mTORC1

The ligand-binding pocket of FRB was an important regulatory site of mTORC1. And FRB sequence of mTORC1 was selected as the receptor protein. Thus, the pocket region where the Rapamycin-FKBP12 complex is bound to inhibit the mTORC1 function was chosen as a reference site. The ZINC15 database provided 17799 purchasable, natural and named product molecules. We selected Rapamycin as a reference compound to assess other compounds’ binding affinity and stability. When the Libdock score of the compound is higher than that of Rapamycin, its docking activity is better [15]. And 7650 compounds were found to have favorable stability when combining with mTORC1 by Libdock algorithm. Additionally, 37 compounds’ Libdock scores were higher than Rapamycin, whose Libdock score was 143.121. Table 1 displays the top 20 ranked compounds following Libdock scores.

**Table 1:** Top 20 ranked compounds with higher libdock scores than Rapamycin.

**Number**	**Compounds**	**Libdock score**	**Number**	**Compounds**	**Libdock score**
1	ZINC000017654900	170.592	11	ZINC000012495776	151.832
2	ZINC000072131515	170.355	12	ZINC000004098458	150.856
3	ZINC000073280937	168.564	13	ZINC000008214470	149.783
4	ZINC000042805482	163.615	14	ZINC000085541163	147.924
5	ZINC000011616633	162.269	15	ZINC000040406945	147.843
6	ZINC000085826837	156.943	16	ZINC000013374324	147.045
7	ZINC000044352341	154.966	17	ZINC000038143593	146.989
8	ZINC000044281738	154.535	18	ZINC000008214697	146.266
9	ZINC000003995616	153.99	19	ZINC000017545457	145.998
10	ZINC000003979028	152.529	20	ZINC000030726863	145.205

### ADME (absorption, distribution, metabolism, excretion) and toxicity prediction

Using the ADME module of DS 4.5, pharmacological properties of Rapamycin and all selected ligands were firstly analyzed, including PPB (plasma protein binding properties), hepatotoxicity, BBB (brain/blood barrier), CYP2D6 (cytochrome P450 2D6) binding, human intestinal absorption and aqueous solubility (Table 2). As results showed, there were different aqueous solubilities (defined in water at 25° C) among different compounds. Compound 1(ZINC000013374324) and compound 2 (ZINC000012495776) had a good solubility. As for human intestinal absorption, Rapamycin and 11 compounds had a low absorption level, and 6 compounds had a poor absorption level. And only one compound had a moderate absorption level. Fortunately, compound 1 and compound 2 had an excellent absorption level. Moreover, most of the compounds were undefined in the Blood-Brain Barrier level except compound 1 and compound 2. Additionally, the results predicted that all compounds are not inhibitors of CYP2D6. Regarding hepatotoxicity, 13 compounds were proved to be nontoxic, similar to Rapamycin. Furthermore, compound 1 and compound 2 were not hepatotoxic and didn’t suppress CYP2D6’s activities. All results above indicated that these two compounds were favorable potential inhibitors of mTORC1. Then, the safety of compounds was also thoroughly tested and evaluated in the following study.

**Table 2:** ADME (Adsorption, Distribution, Metabolism, Excretion) properties of compounds.

**Number**	**Compounds**	**Solubility level^a^**	**BBB level^b^**	**CYP2D6^c^**	**Hepatotoxicity^d^**	**Absorption level^e^**	**PPB level^f^**
1	ZINC000017654900	2	4	0	1	2	0
2	ZINC000072131515	0	4	0	0	3	1
3	ZINC000073280937	2	4	0	1	2	1
4	ZINC000042805482	2	4	0	0	2	0
5	ZINC000011616633	2	4	0	0	3	0
6	ZINC000085826837	2	4	0	0	2	0
7	ZINC000044352341	4	4	0	0	3	0
8	ZINC000044281738	0	4	0	1	3	1
9	ZINC000003995616	1	4	0	0	2	1
10	ZINC000003979028	2	4	0	1	3	0
11	ZINC000012495776	4	3	0	0	0	1
12	ZINC000004098458	3	4	0	0	3	0
13	ZINC000008214470	1	4	0	1	3	0
14	ZINC000085541163	2	4	0	0	2	0
15	ZINC000040406945	2	4	0	0	1	0
16	ZINC000013374324	2	2	0	0	0	0
17	ZINC000038143593	3	4	0	0	3	0
18	ZINC000008214697	2	4	0	0	3	1
19	ZINC000017545457	4	4	0	1	3	0
20	ZINC000030726863	0	4	0	1	3	0
21	Rapamycin	3	4	0	0	3	1

^a^Aqueous-solubility level: 0 (extremely low); 1 (very low, but possible); 2 (low); 3 (good).

^b^Blood Brain Barrier level: 0 (Very high penetrant); 1 (High); 2 (Medium); 3 (Low); 4 (Undefined).

^c^Cytochrome P450 2D6 level: 0 (Non-inhibitor); 1 (Inhibitor).

^d^Hepatotoxicity: 0 (Nontoxic); 1 (Toxic).

^e^Human-intestinal absorption level: 0 (good); 1 (moderate); 2 (poor); 3 (very poor).

^f^Plasma Protein Binding: 0 (Absorbent weak); 1 (Absorbent strong).

**Figure 2:**
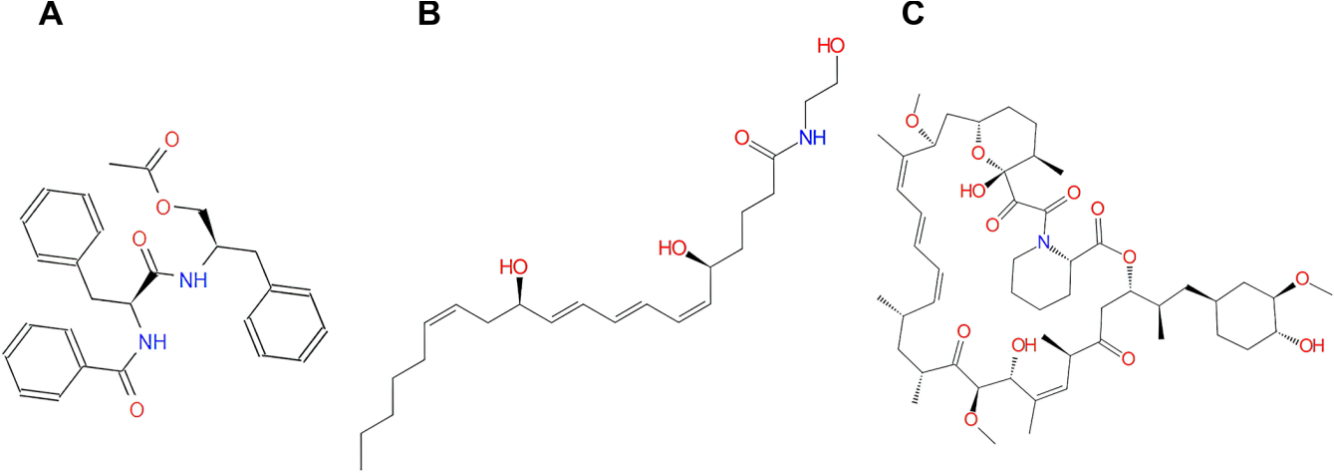
**The structures of Rapamycin and novel compounds selected from virtual screening.** (**A**) ZINC000013374324; (**B**) ZINC000012495776; (**C**) Rapamycin.

To examine the safety of the top 20 ranked compounds, several toxicity indicators of Rapamycin and the compounds were predicted with TOPKAT module of DS 4.5 (Table 3), including AMES (Ames mutagenicity), DTP (developmental toxicity potential) properties and Rodent carcinogenicity (based on the U.S. National Toxicology Program (NTP) dataset). Moreover, in contrast with other compounds, compound 1 and compound 2 were predicted with less developmental toxicity, rodent carcinogenicity and AMES mutagenicity according to the prediction, indicating their perspective application in inhibitor-drug development of mTORC1. As shown in Figure 2, Rapamycin and compound 1, 2 were quite similar for their chemical constructions, containing several dual-band and multiple reactive oxygens in chemical structure. More importantly, both Rapamycin and these two compounds bind to mTORC1 in the same position. In conclusion, compounds 1, 2 were proved safe and chosen for follow-up study (Figure 2).

**Table 3:** Toxicities of compounds.

**Number**	**Compounds**	**Mouse NTP^a^**		**Rat NTP^a^**		**AMES^b^**	**DTP^c^**
		**Female**	**Male**	**Female**	**Male**		
1	ZINC000017654900	0.5712	0.3802	0.2197	0.3239	0.0328	0.4466
2	ZINC000072131515	0.5721	0.0048	0.1620	0.5175	0.0000	0.3214
3	ZINC000073280937	0.6259	0.5248	0.3603	0.2711	0.0002	0.5399
4	ZINC000042805482	0.8020	0.8727	0.4764	0.2905	0.0118	0.5020
5	ZINC000011616633	0.4438	0.3649	0.3071	0.1586	0.0968	0.8155
6	ZINC000085826837	0.7606	0.5090	0.3085	0.5828	0.0000	0.4933
7	ZINC000044352341	0.4438	0.3649	0.3071	0.1586	0.0968	0.8155
8	ZINC000044281738	0.4447	0.6051	0.1954	0.2057	0.2047	0.8230
9	ZINC000003995616	0.5496	0.8186	0.2477	0.3085	0.1945	0.5723
10	ZINC000003979028	0.2346	0.0025	0.2452	0.2996	0.0042	0.2524
11	ZINC000012495776	0.4786	0.4815	0.4937	0.7482	0.5109	0.6602
12	ZINC000004098458	0.4905	0.2701	0.1576	0.1179	0.0260	0.3511
13	ZINC000008214470	0.5908	0.5543	0.3714	0.4447	0.1682	0.6034
14	ZINC000085541163	0.5261	0.3273	0.3300	0.6175	0.0000	0.6446
15	ZINC000040406945	0.4438	0.3649	0.3071	0.1586	0.0968	0.8155
16	ZINC000013374324	0.6211	0.4172	0.2620	0.4974	0.0055	0.5064
17	ZINC000038143593	0.2039	0.5345	0.4413	0.4888	0.1035	0.5153
18	ZINC000008214697	0.3840	0.4048	0.2651	0.3001	0.1780	0.6137
19	ZINC000017545457	0.6418	0.5990	0.2244	0.3102	0.0577	0.4543
20	ZINC000030726863	0.4132	0.0579	0.2043	0.2855	0.4225	0.4626
21	Rapamycin	0.5536	0.6142	0.4147	0.5873	0.9970	0.6185

^a^<0.3 (Non-Carcinogen); >0.7 (Carcinogen).

^b^<0.3 (Non-Mutagen); >0.7 (Mutagen).

^c^<0.3 (Non-Toxic); >0.7 (Toxic).

### Ligand binding and analysis and pharmacophore

**Figure 3:**
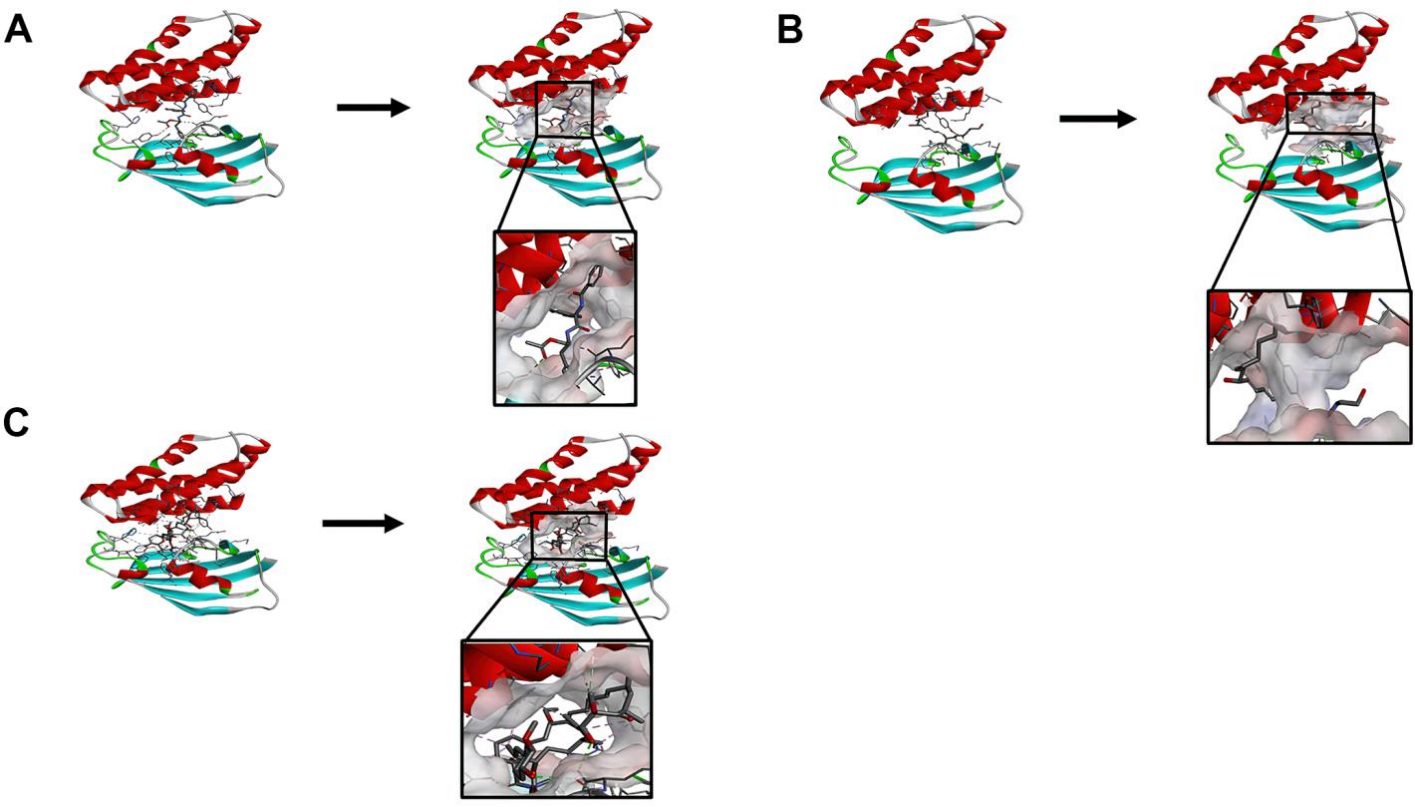
(**A**) ZINC000013374324-mTORC1 complex. Schematic drawing of interactions between ligands and mTORC1, the surface of the binding area was added, blue represented positive charge, red represented negative charge, and ligands were shown in the sticks, the structure around the ligand-receptor junction was shown in thinner sticks. (**B**) ZINC000012495776 -mTORC1 complex. Schematic drawing of interactions between ligands and mTORC1, the surface of the binding area was added, blue represented positive charge, red represented negative charge, and ligands were shown in the sticks, the structure around the ligand-receptor junction was shown in thinner sticks. (**C**) Rapamycin-mTORC1 complex. Schematic drawing of interactions between ligands and mTORC1, the surface of the binding area was added, blue represented positive charge, red represented negative charge, and ligands were shown in the sticks, the structure around the ligand-receptor junction was shown in thinner sticks.

**Figure 4:**
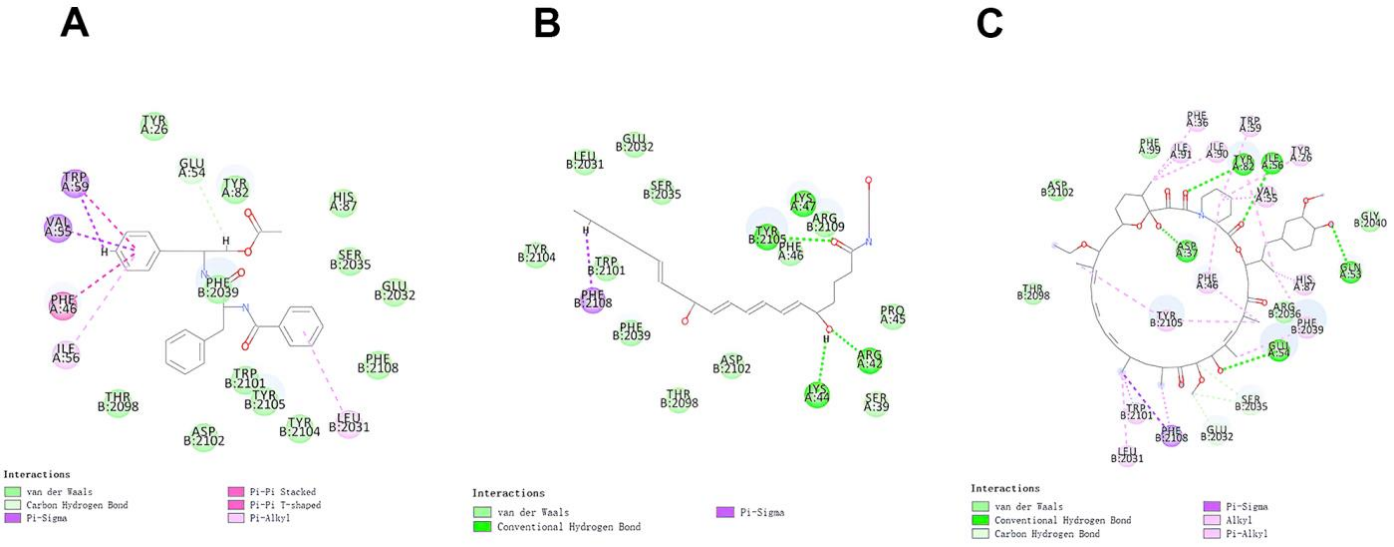
The inter-molecular interaction of the predicted binding modes of (**A**) ZINC000013374324 to mTORC1; (**B**) ZINC000012495776 to mTORC1.; (**C**) Rapamycin to mTORC1.

The RMSD (Root Mean Square Deviation) between the docked pose and the crystal structure of the complex was 0.6 Å, which indicated that the application of the CDOCKER module was relatively reliable in this study. Under the CHARMm36 force field, two selected compounds were docked into mTORC1’s pocket site by the CDOCKER module. The calculation of CDOCKER potential energy was displayed in Tables 4, 5. Moreover, the CDOCKER Interaction energy of the reference ligand Rapamycin (-46.4464kcal/mol) was higher than that of compound 1, compound 2, indicating that these two compounds may have a higher binding affinity with mTORC1 in contrast with Rapamycin. Structural analysis was conducted for the Pi-Pi interactions and hydrogen bonds of ligand-mTORC1 complexes (Figures 3, 4 and Table 6). Results demonstrated that compound 2 had four pairs of hydrogen bonds with mTORC1, through the O23 of compound and B: TYR2105:HH of mTORC1, the O23 of compound and A: LYS47:HN of mTORC1, the O18 of compound and A: ARG42:HH21 of mTORC1, the H52 of compound and A: LYS44:O of mTORC1. Besides, only one pair of Pi-Sigma interaction was found in the compound 2- mTORC1 complex. Compound 1 didn’t form a hydrogen bond with mTORC1. However, 6 pairs of pi interactions formed in the compound 2- mTORC1 complex, including two pairs of Pi-Sigma interaction, two pairs of Pi-Pi interaction, and two pairs of Pi-Alkyl interaction. Regarding Rapamycin, four hydrogen bonds (A:ASP37:OD2:A: ARD108:H1, A: GLN53:O:A: ARD108:H3, A:TYR82:HH:A: ARD108:O1, A:ILE56:HN:A: RAD108:O2, respectively) were formed with mTORC1. Additionally, Rapamycin formed fifteen pairs of pi interactions with mTORC1, including one pair of Pi-Sigma interaction and fourteen Pi-Alkyl interactions.

**Table 4:** CDOCKER interaction energy of compounds with mTORC1.

**Compounds**	**CDOCKER interaction energy (Kcal/mol)**
ZINC000013374324	-49.0963
ZINC000012495776	-47.1762
Rapamycin	-46.4464

**Table 5:** Hydrogen bond interaction parameters for each compound and mTORC1 residues.

**Receptor**	**Compound**	**Donor atom**	**Receptor atom**	**Distances (Å)**
mTORC1	ZINC000012495776	B:TYR2105:HH	ZINC000012495776:O23	2.42
		A:LYS47:HN	ZINC000012495776:O23	1.74
		A:ARG42:HH21	ZINC000012495776:O18	1.83
		A:LYS44:O	ZINC000012495776:H52	2.45
	Rapamycin	A:ASP37:OD2	A:ARD108:H1	1.69
		A:GLN53:O	A:ARD108:H3	1.65
		A:TYR82:HH	A:ARD108:O1	2.92
		A:ILE56:HN	A:RAD108:O2	1.97

**Table 6:** Pi-Sigma interaction, Pi-Pi interaction, Pi-Alkyl interaction and Alkyl interaction parameters for each compound and mTORC1 residues.

**Interaction parameters**	**Receptor**	**Compound**	**Donor atom**	**Receptor atom**	**Distances (Å)**
Pi-Sigma interaction	mTORC1	ZINC000013374324	A:TRP59	ZINC000013374324:H44	2.64
			A:VAL55:CG1	ZINC000013374324	3.94
		ZINC000012495776	B:PHE2108	ZINC000012495776:H31	2.75
		Rapamycin	B:PHE2108	A:RAD108:C44	3.74
Pi-Pi interaction		ZINC000013374324	A:TRP59	ZINC000013374324	5.04
			A:TRP59	ZINC000013374324	5.08
Pi-Alkyl interaction		ZINC000013374324	A:ILE56	ZINC000013374324	5.41
			B:LEU2031	ZINC000013374324	5.33
		Rapamycin	B:PHE2108	A:RAD108:C45	5.28
			B:TRP2101	A:RAD108:C44	5.46
			B:TYR2105	A:RAD108:C47	4.61
			B:TYR2105	A:RAD108:C43	4.42
			A:PHE46	A:RAD108:C47	5.18
			A:PHE46	A:RAD108	4.72
			A:TRP59	A:RAD108	4.19
			A:TRP59	A:RAD108	4.56
			A:TYR26	A:RAD108	4.89
			A:PHE36	A:RAD108:C42	4.47
			A:TYR82	A:RAD108:C48	5.09
			A:HIS87	A:RAD108:C48	4.65
			B:PHE2039	A:RAD108:C48	4.59
			B:PHE2039	A:RAD108:C46	4.34
Alkyl interaction		Rapamycin	B:LEU2031	A:RAD108:C44	4.78
			A:VAL55	A:RAD108	5.35
			A:ILE91	A:RAD108:C42	4.78
			A:ILE90	A:RAD108:C42	4.82

**Figure 5:**
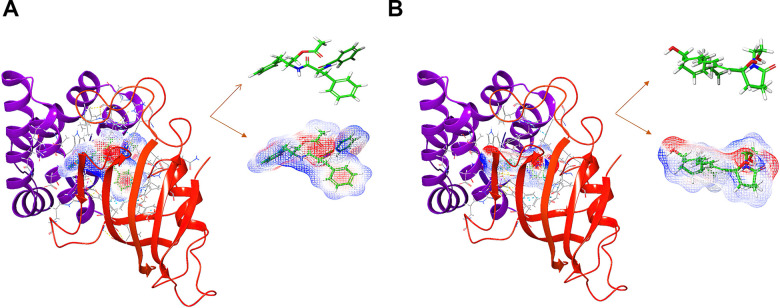
**The molecular docking by Schrodinger.** Ligands were docked into the defined binding pocket. Red represents positive charge; blue represents negative charge. (**A**) ZINC000013374324 to mTORC1; (**B**) ZINC000012495776 to mTORC1.

**Figure 6:**
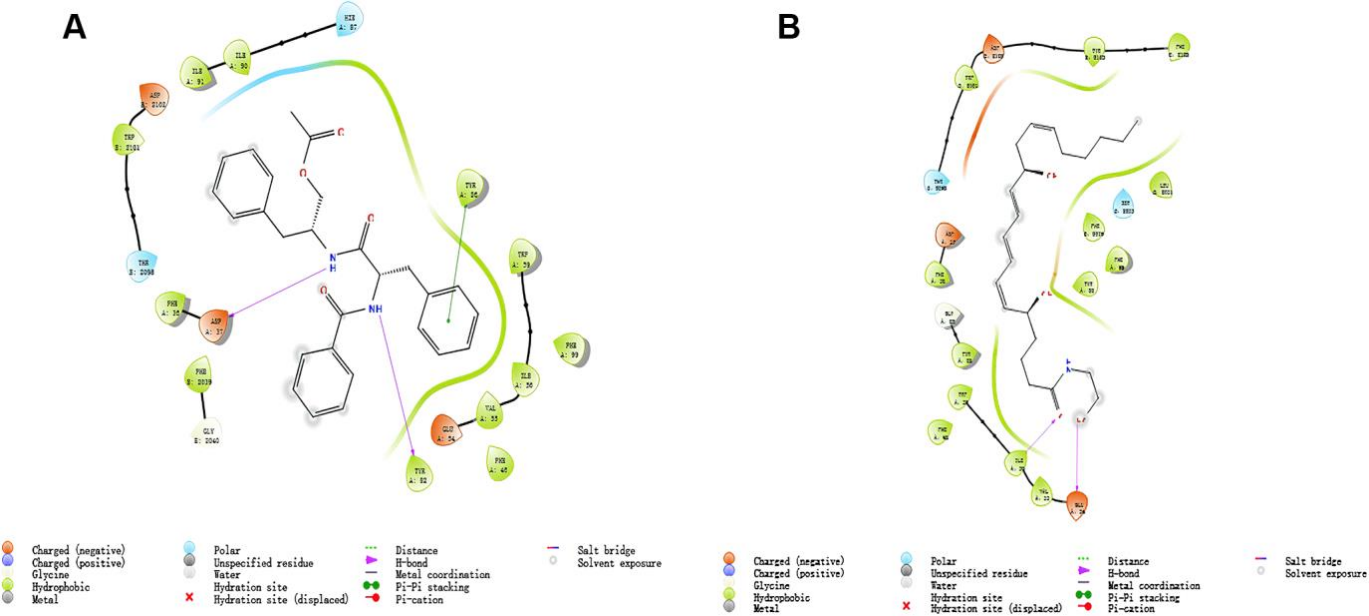
The inter-molecular interaction by Schrodinger of the predicted binding modes of (**A**) ZINC000013374324 to mTORC1; (**B**) ZINC000012495776 to mTORC1.

Additionally, to ensure the credibility of the results carried out with CDOCKER, the results were crosschecked again through Schrodinger. All docking conformations were visualized to ensure the docking at the designated place. The 3D structures of compound 1-mTOR complex and compound 2-mTOR complex are shown in Figure 5. The interactions between compound 1-mTOR complex and compound 2-mTOR complex were shown in Figure 6.

**Figure 7:**
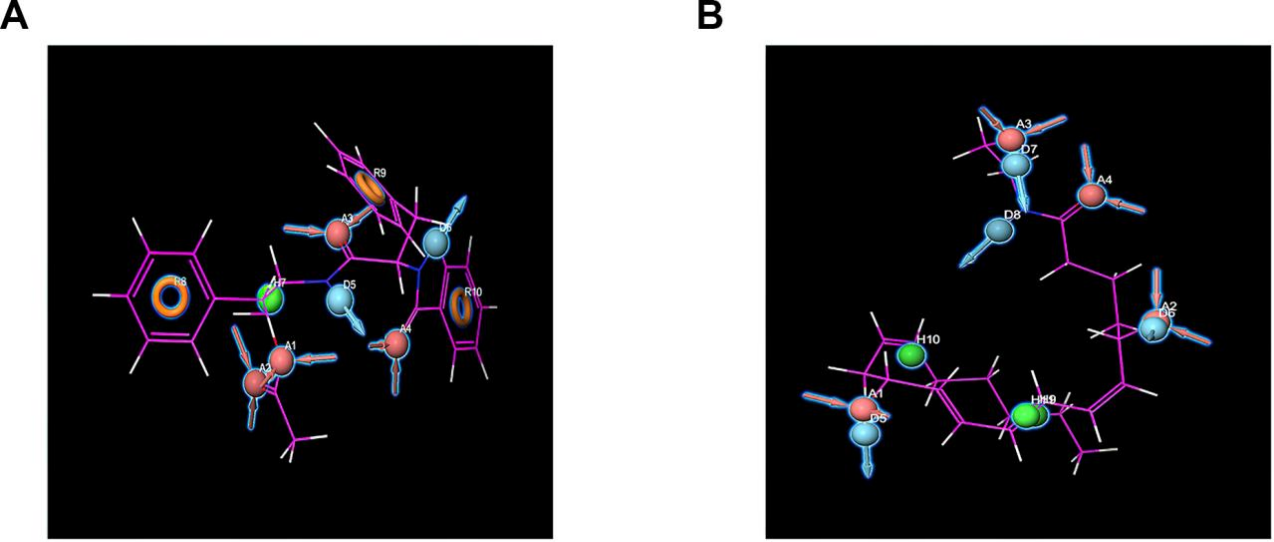
**Pharmacophore predictions using Schrodinger.** Red represents hydrogen acceptor; blue represents hydrogen donor, green represents the hydrophobic center, and yellow represents Aromatic Ring. (**A**) ZINC000013374324 to mTORC1; (**B**) ZINC000012495776 to mTORC1.

Furthermore, the pharmacophore part of the result has also been supplemented by Schrodinger, such as the pharmacophore of small molecules in the docking conformation with the protein (Figure 7). Computation results showed 10 feature pharmacophores in ZINC000012495776 and 11 feature pharmacophores in ZINC000013374324. ZINC000012495776 had four hydrogen acceptors, two hydrogen donors, one hydrophobic center, and three Aromatic Rings. ZINC000013374324 had four hydrogen acceptors, four hydrogen donors, three hydrophobic centers.

### Molecular dynamics simulation

**Figure 8:**
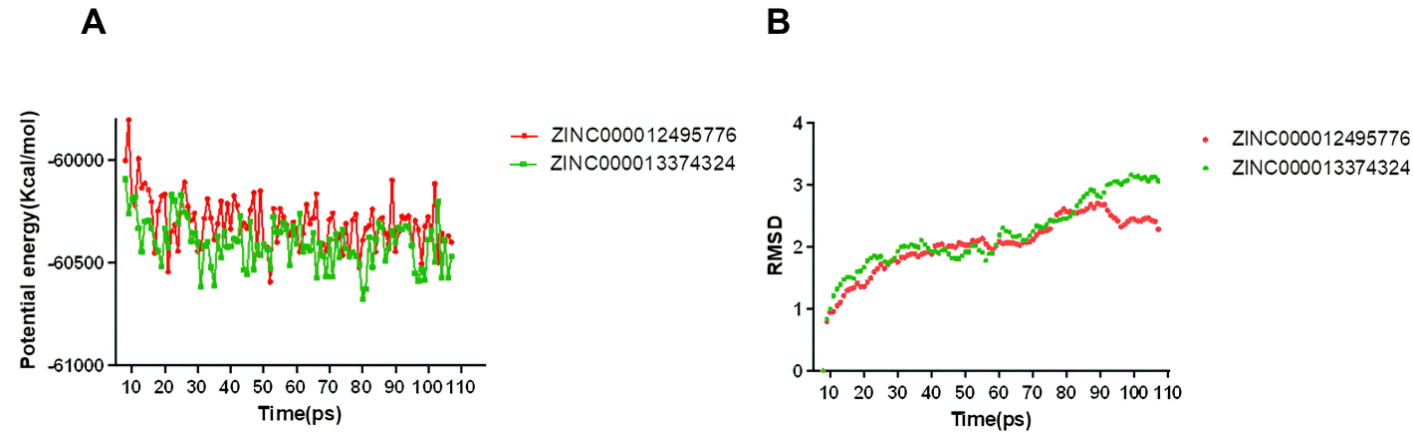
**Results of molecular dynamics simulation of three complexes.** (**A**) Potential energy; (**B**) Average backbone RMSD.

We performed the molecular dynamics simulation module to assess if the ligand-mTORC1 complexes were stable under natural environment circumstances. Dynamic analysis is based on the molecular force field, which can dynamically describe the motion of molecules. It mainly analyzed the potential energy and RMSD of the protein-ligand complex. The original conformations were obtained in the molecular docking experiment through the CDOCKER module. The potential energy and RMSD curves chart of ligand-mTORC1 complexes were shown in Figure 8. The two curves finally tend to be stable. Based on the results of Libdock and CDOCKER, when the score of Libdock and the absolute value of CDOCKER potential energy are higher, ligand and protein bind dynamically better. The trajectories of each complex reached equilibrium after 90 ps. RMSD and the potential energy of these complexes got stable over time. The results verified that these pi-related interactions and hydrogen bonds formed by compounds 1, 2 and mTORC1 promote the stability of these complexes. Finally, we could conclude that their complexes stably exist in the natural environment, and as mTORC1 inhibitors have a regulatory effect on mTORC1.

### Establishment of the enzymatic reaction system and determination of mTOR protein activity

**Figure 9:**
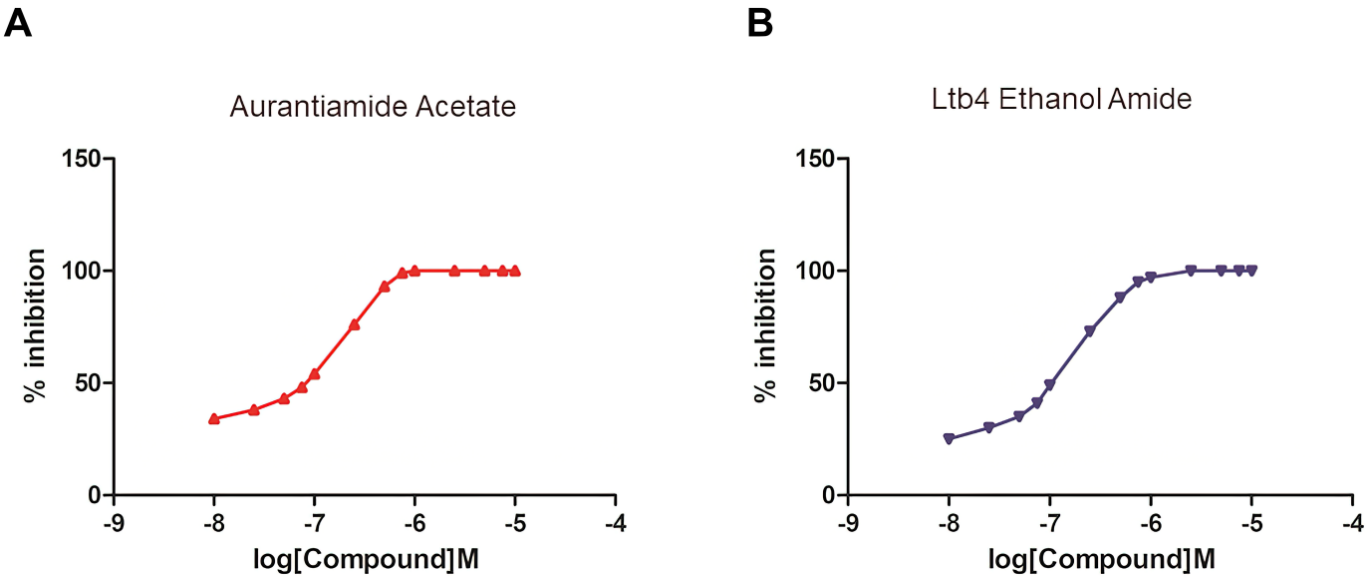
**The establishment of an enzymatic reaction system of different concentrations of selected molecules and the determination of mTOR protein activity.** (**A**) Aurantiamide Acetate; (**B**) Ltb4 Ethanol Amide.

Finally, an enzymatic reaction experiment of the mTOR protein was carried out to verify our conclusion. mTOR promotes the activation of Atg13 protein phosphorylation. Two selected compounds at different concentrations were used to detect the degree of inhibition of mTOR by calculating substrate, namely Atg13’s phosphorylation inhibition rate at different concentrations. The results showed that with the increase of drug concentration, the inhibition degree of Atg13 was more substantial (Figure 9). In addition, the experimental results showed that under the experimental conditions set by us, the two drugs could completely inhibit the substrate of mTOR at about 10umol/L. Therefore, as the concentration of two selected drug concentrations increases, the activity of mTOR protein was continuously inhibited. And when the drug concentration was 10nmol/l, the inhibitory effect was almost complete.

## DISCUSSION

Ischemic stroke has the four characteristics of high prevalence, high disability, high mortality and high recurrence rate. There have been rapid advances in the treatment of ultra-early thrombolysis and thrombectomy for ischemic stroke and improving collateral circulation in the acute phase. The death rate and disability rate of stroke have dropped significantly. However, the early and ultra-early blood reperfusion involves reperfusion injury, which will cause secondary nerve damage and is called CIRI. Thus, CIRI is an essential factor that aggravates the pathophysiological process of cerebral ischemia prognosis.

Moreover, studies have found that autophagy is involved in the entire process of CIRI [21]. As an adaptive cellular response, autophagy tries its best to maintain cell homeostasis by removing misfolded proteins and damaged organelles so that cells can avoid apoptosis. Autophagy is a hot spot in biomedical research. And it is a process of lysosome-mediated degradation of cellular components. When the number of damaged organelles increases, external pathogens invade or abnormal accumulation of proteins, cell contents will be wrapped in the vesicle membrane structure to form autophagosomes and then integrate with lysosomes to form autolysates [22, 23]. Then, the cell content will be degraded into small molecules that can undergo aerobic respiration [23]. In 1995, Nitatori et al. used transmission electron microscopy to confirm the occurrence of autophagy in nerve cells after cerebral ischemia for the first time [22]. More and more evidence has shown that brain CIRI injury has a close relationship to autophagy [24]. Research by Zhang et al. shows that Astragaloside IV therapy protects the brain from CIRI damage by promoting autophagy [25]. Autophagy activation induced by LncRNA SNHG12 reduced brain CIRI damage, and autophagy inhibitor 3-MA partially reversed this damage [26]. All these studies indicate that autophagy exerts a neuroprotective effect after brain I/R injury.

In addition, the mammalian target of Rapamycin (mTORC1) is the primary signal pathway regulating autophagy. mTORC1 inhibitors can prevent anti-apoptotic signals, thereby stimulating autophagy and inhibiting apoptosis from exerting neuroprotective effects [10, 27]. Furthermore, mTORC1 inhibitors can inhibit microglial activation and reduce the release of neuroinflammatory mediators, which will protect the penumbra after CIRI from secondary damage [11, 12]. What’s more, the mTORC1 inhibitor, Rapamycin, has been proven to exert neuroprotective effects in the ultra-early and early cerebral ischemia-reperfusion. Hence, screening and designing mTORC1 inhibitors are essential to improve the functional recovery of patients with cerebral ischemia by controlling CIRI, reducing neuronal death and apoptosis. In addition, although some of the existing drugs have been shown to play a neuroprotective effect on ischemia and hypoxia injury in animal models and *in vitro* experiments, they are clinically ineffective. So, developing new treatment methods or drugs targeting the mTORC1 protein in the autophagy pathway is particularly important for reducing and treating CIRI [3]. Rapamycin can bind to FKBP12 and inhibit mTORC1, thereby activating autophagy and immunosuppression. Therefore, Rapamycin was selected as the reference molecule for mTORC1 inhibitors. And FRB sequence was positioned as the binding site of protein inhibitor for a series of inhibitor screening.

Furthermore, novel potential compounds’ structural and biological properties were screened and analyzed by five modules of DS 4.5 and two modules of Schrodinger [27]. Toxicological properties, pharmacological properties, molecular conformation, binding stability and affinity were also thoroughly calculated to identify superior compounds. From the ZINC15 database, we obtained 17799 named, natural and purchasable product molecules for virtual screening. The top 20 molecules were picked out in accordance with Libdock score and used for follow-up research. Libdock score was an indicator of conformational stability and energy optimization. Compounds with a high Libdock score reflected their stable conformations and pretty energy optimizations in contrast with others. According to the calculation of DS 4.5’s Libdock module, 7650 molecules had a high binding affinity with mTORC1. Moreover, Libdock scores of 37 molecules were higher than the reference compound Rapamycin, indicating that these 37 compounds could combine with mTORC1 well and form a better energy optimization with more stable conformation in contrast with Rapamycin.

In addition, ADME and toxicity properties were conducted to assess the pharmacological and toxicological properties of these chosen molecules. Results demonstrated that compound 1 (ZINC000013374324) and compound 2 (ZINC000012495776) were identified as favorable inhibitors of mTORC1. The reason is as follows. First of all, compound 1 and compound 2 were soluble and also had an excellent absorption level. And both two selected compounds were not hepatotoxic and non-inhibitors of CYP2D6. Additionally, in contrast with other compounds, they were predicted with less developmental toxicity potential, rodent carcinogenicity and AMES mutagenicity, suggesting that they can be applied in drug development. Furthermore, there are also potential applications of other small molecules in the list in drug development. Even though their current structure was toxic, we could add specific groups and atoms to reduce their toxicity. Considering all the above, we selected compounds 1, 2 as favorable inhibitors of mTORC1 and for further analyses.

Moreover, the investigation was also performed over the chemical bonds and binding mechanism of the chosen candidate compound 1, 2. It is pretty clear that the CDOCKER interaction energy of the two compounds, according to CDOCKER module computation, was obviously lower than the reference ligand Rapamycin (-46.4464kcal/mol). Next, the chemical structures and binding mechanisms of these compounds were analyzed in this study. Results indicated that these compounds could contain several carbon-carbon double bonds and carbon-oxygen double bonds, similar to Rapamycin. So, this is why they could connect with mTORC1. Then, Schrodinger has applied to re-docking the mTORC1 protein with two selected molecules to ensure the credibility of the results carried out with CDOCKER. In addition, we also analyzed the feature pharmacophores of these two compounds in the docking conformation with the protein. And the pharmacophores of compounds 1, 2 were displayed.

In this module, the potential energy and RMSD of these ligand-mTORC1 complexes were analyzed. Firstly, the results show that it took 90ps for the trajectory of the complex to reach equilibrium. Secondly, the potential energy and RMSD of the complexes gradually got stabilized over time. This case showed that these two complexes could exist stably in the natural environment. What’s more, by performing the molecular dynamic simulation, their stabilities were also thoroughly evaluated. Based on the results above, modifications and improvements can be made to make the ligand and receptor bind more firmly. What is noteworthy is that the compounds studied in our research mainly focused on developing inhibitors. Featuring their innate affinity for mTORC1, natural compounds identified during the research might be a potentially valuable resource for developing mTORC1 related drugs [28].

Additionally, an enzymatic reaction experiment of the mTOR protein was performed to verify the effects of potential mTOR inhibitors. As we all know, mTOR promotes the activation of Atg13 protein, that is, phosphorylation. So, we applied two selected compounds at different concentrations to detecting the degree of inhibition of mTOR by calculating substrate, namely Atg13’s phosphorylation inhibition rate. As the results show, with the concentration of two selected drugs’ concentrations increasing, activity of mTOR protein was continuously inhibited. And when the drug concentration was 10nmol/l, the inhibitory effect was almost complete. Therefore, Aurantiamide Acetate and Ltb4 Ethanol Amide were proved to be ideal inhibitors of mTORC1.

Last but not least, this study attempted to find more favorable mTORC1 inhibitors to significantly promote the development of mTORC1 related CIRI therapeutic drugs. Despite the elaborate design and accurate measurements, it is hard to deny that there are still a few limitations in this study. More experiments *in vivo* can be carried out in the future to validate our results. And Aerobic Biodegradability (A.B.) and Maximum Tolerated Dosage (MTD) measurements can be calculated regarding drug safety in our future study.

## CONCLUSIONS

In this study, a series of structural biology and chemical methods (including virtual screening, molecular docking, etc.) were used to screen and identify lead compounds with potential inhibitory function to mTORC1. In summary, compounds 1 and 2 were safe drug candidates and could significantly promote mTORC1-related CIRI therapeutic drug development. In addition, a list of drug candidates with pharmacological properties was provided, laying a solid foundation for the development and research of mTORC1 inhibitors.
